# Multifaceted functions of RNA-binding protein vigilin in gene silencing, genome stability, and autism-related disorders

**DOI:** 10.1016/j.jbc.2023.102988

**Published:** 2023-02-08

**Authors:** Arjamand Mushtaq, Ulfat Syed Mir, Mohammad Altaf

**Affiliations:** Centre for Interdisciplinary Research and Innovations, University of Kashmir, Srinagar, Jammu and Kashmir, India

**Keywords:** RNA-binding proteins, gene silencing, heterochromatin, genome organization, autism-related disorders, ASD, autism spectrum disorder, CTCF, CCCTC-binding factor, DDP1, Drosophila dodeca-satellite-binding protein 1, DSB, double-stranded break, FMR1, fragile X mental retardation 1 protein, HCC, hepatocellular carcinoma, HR, homologous recombination, HuR, human antigen R, IR, ionizing radiation, KH, K homology, NAFLD, nonalcoholic fatty liver disease, NPC, neural progenitor cell, RBP, RNA-binding protein, SCLC, small cell lung cancer

## Abstract

RNA-binding proteins (RBPs) are emerging as important players in regulating eukaryotic gene expression and genome stability. Specific RBPs have been shown to mediate various chromatin-associated processes ranging from transcription to gene silencing and DNA repair. One of the prominent classes of RBPs is the KH domain–containing proteins. Vigilin, an evolutionarily conserved KH domain–containing RBP has been shown to be associated with diverse biological processes like RNA transport and metabolism, sterol metabolism, chromosome segregation, and carcinogenesis. We have previously reported that vigilin is essential for heterochromatin-mediated gene silencing in fission yeast. More recently, we have identified that vigilin in humans plays a critical role in efficient repair of DNA double-stranded breaks and functions in homology-directed DNA repair. In this review, we highlight the multifaceted functions of vigilin and discuss the findings in the context of gene expression, genome organization, cancer, and autism-related disorders.

RNA-binding proteins (RBPs) control diverse cellular functions and act as regulatory factors in gene expression, genome stability, mRNA transport and metabolism, RNA stability, and development of immune system (Fig. 1) (1). RBPs also regulate proteosome diversity through alternative splicing. A single RBP can bind and regulate multiple mRNA species and thus have a considerable influence over cell function (2). RBPs contain one or multiple RNA-binding protein domains like RNA Recognition Motif, RGG (Arg-Gly-Gly) box, K homology (KH) domain, zinc finger, Pumilio/PUF domain, double-stranded RNA-binding domain, and Piwi/Argonaute/Zwille domain (3, 4). One of the sequence-specific RBPs is the Pumilio/PUF domain family that interact with mRNA and regulate translation (5). In *Saccharomyces cerevisiae* RBPs CTH1 (Cysteine three histidine protein 1) and CTH2 having zinc finger CCCH domains are involved in mRNA stability and iron homeostasis (6). MSN2 (Multicopy Suppressor of SNF1 mutation) and MSN4 act as transcriptional activators under stress conditions and activate genes involved in protective response (7, 8). RBPs associate with nascent RNA transcripts and help in recruitment of the multimeric ribonucleoprotein complex, spliceosome that edits the nascent RNAs nucleotide sequence by removing intronic regions (9, 10, 11). The RBP TRBP (transactivation response element RNA-binding protein), a molecular chaperone, is required for DICER1 function, and depletion or loss of function of TRBP results in abnormal expression of miRNA and cancer cell proliferation and differentiation (12). In neurons, RBPs are involved in delivery of specific mRNAs to different subcellular compartments to be locally translated. RBPs regulate neuronal function by trafficking selective mRNAs and regulating local protein synthesis and determine axonal or dendritic mRNA repertoires (13). Besides their role in regulating gene expression and RNA processing, RBPs have been implicated in a number of diseases and disorders when dysregulated or mutated (14, 15). Depletion or mutations in genes encoding RBPs like TDP-43 (TAR DNA-binding protein 43), FUS (fused in sarcoma), and FMRP (fragile X mental retardation protein) leads to human neurological disorders such as spinal muscular atrophy, amyotrophic lateral sclerosis, and fragile X syndrome (16).

**Figure 1:**
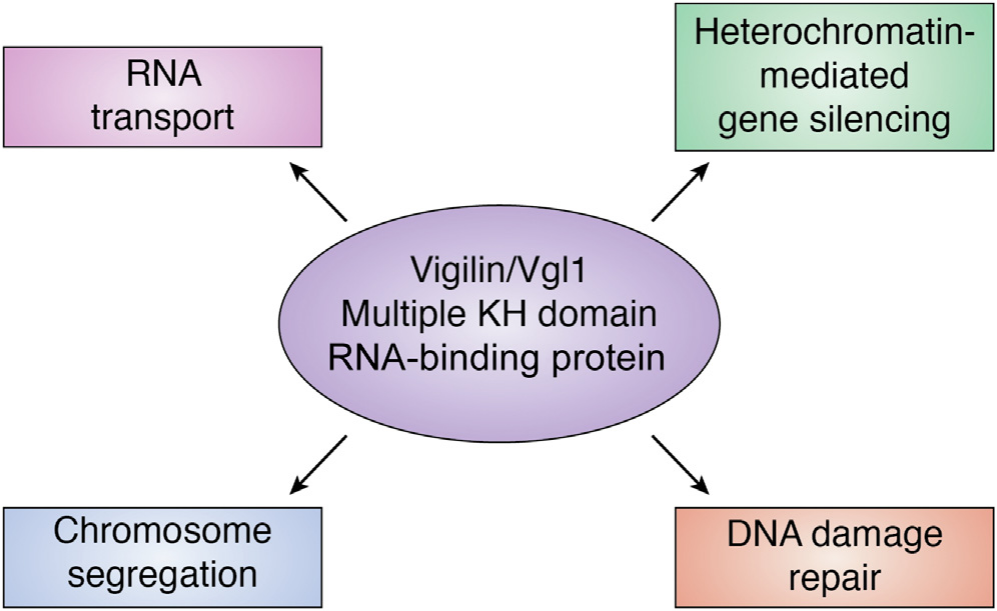
**Diverse functions of KH domain–containing protein vigilin**.

One of the important classes of RBPs is the KH domain–containing proteins. The KH domain is an evolutionarily conserved domain first identified in the heteronuclear ribonucleoprotein particle (hnRNP) K as a repeat sequence (17). KH domains are composed of 70 to 100 amino acid residues with a conserved GxxG motif (18). KH domains can mediate protein–protein and protein–nucleic acid interactions and regulate the function of these RBPs (19, 20). Vigilin also known as high-density lipoprotein–binding protein is the largest RBP of KH domain–containing family of proteins and contains 14 tandemly arranged nonidentical type hnRNP KH domains (19, 20). Vigilin is evolutionarily conserved from yeast *S. cerevisiae* Scp160 (RBP containing 14 KH domains), *Schizosaccharomyces pombe* (Vgl1) to *Drosophila* (DDP1, *Drosophila* dodeca-satellite-binding protein 1) and vertebrates (Vigilin) and has been shown to be associated with diverse biological processes like RNA transport and metabolism, sterol metabolism, chromosome segregation, heterochromatin formation, DNA repair, and carcinogenesis (19, 21, 22, 23). Vigilin has been shown to interact with CCCTC-binding factor (CTCF) and regulates insulin-like growth factor 2 (Igf2) and H19 imprinted genes (24). Igf2 is expressed from the paternal allele and H19 from the maternal allele (25). Furthermore, vigilin has been shown to be associated with autism and brachymetaphalangy, where 2q37 deletion syndrome results in downregulation of vigilin and causes the Albright hereditary osteodystrophy-like phenotype, characterized by developmental delay, mental retardation, and brachymetaphalangy (26, 27). Despite the well-defined roles of vigilin in various cellular processes, we still do not have a full understanding of how various functions of vigilin are mechanistically regulated. In this review, we present the recent findings about vigilin in context to gene silencing, genome stability, carcinogenesis, and autism-related disorders.

## Role of vigilin in heterochromatin gene silencing

**Figure 2:**
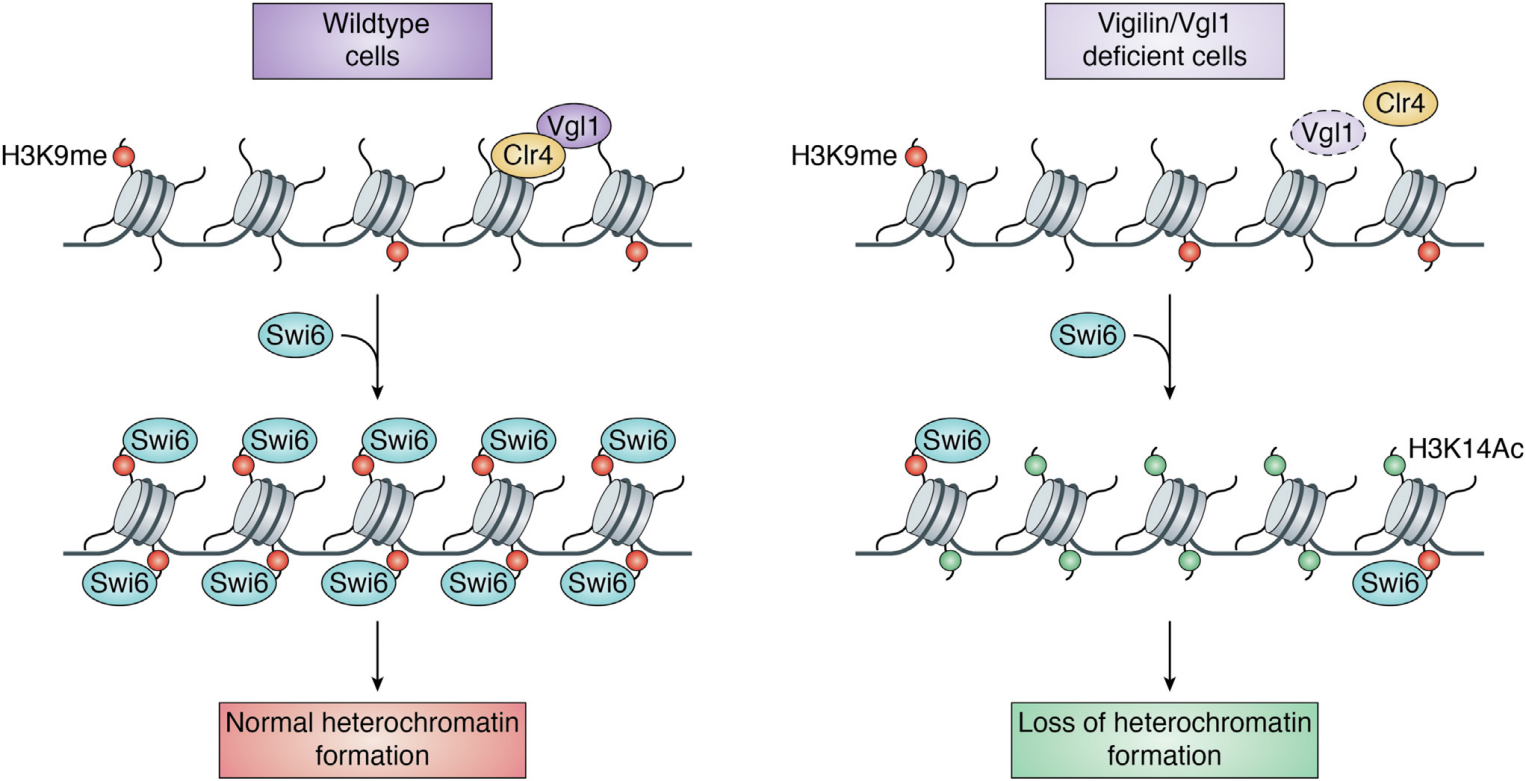
**Vigilin in Gene Silencing.** Vigilin gets recruited to pericentromeric heterochromatin in an RNA-dependent manner and helps in recruitment of Clr4, which leads to H3K9 methylation and Swi6 recruitment. This sequence of events is essential for proper heterochromatin formation.

Eukaryotic genome is organized into a nucleoprotein complex called chromatin, which regulates all DNA-mediated processes. Nucleosome, the fundamental unit of chromatin, is composed of histone proteins comprising two copies each of H2A, H2B, H3, and H4 (28, 29). Chromatin is organized into euchromatin, which is transcriptionally active, gene rich, and loosely packaged, and heterochromatin, which is highly condensed, transcriptionally inactive, and gene poor (28, 30). Furthermore, these chromatin domains also differ in the nature of histone posttranslational modifications, which in turn decide chromatin compaction and the type of proteins that recognize these modifications. Euchromatin is rich in histone acetylation and H3K4 methylation, whereas heterochromatin is hypoacetylated and rich in repressive H3K9 methylation and H4K20 trimethylation (28, 30). Heterochromatin organization regulates processes like gene expression and chromosome segregation and is crucial for maintaining genomic stability (31, 32). One of the defining features of heterochromatin organization in many eukaryotes is the methylation of H3K9, which acts as a binding site for heterochromatin protein 1 (HP1/Swi6) (33, 34). Apart from H3K9 methylation and HP1, several other proteins have been shown to be important for heterochromatin gene silencing (33, 34). Vigilin is one such protein that has been shown to be associated with heterochromatin gene silencing across species (35, 36). In *S. cerevisiae*, loss of vigilin causes cell ploidy and silencing defects at telomeres and mating type loci (37, 38, 39). However, the precise molecular mechanism of how vigilin regulates ploidy and silencing in *S. cerevisiae* is not known. In *S. pombe*, vigilin has been shown to be indispensable for heterochromatic gene silencing at centromeres and telomeres (40). Vgl1 binds to heterochromatic domains in an RNA-dependent manner, and loss of Vgl1 impairs H3K9 methylation and Swi6 recruitment to chromatin (40). Mechanistically, Vgl1 interacts with H3K9 methyltransferase, Clr4, and helps in its recruitment to chromatin. Deletion of Vgl1 impairs Clr4 recruitment and loss of H3K9 methylation at centromeric and telomeric heterochromatin regions (Fig. 2) (40). In *Drosophila*, vigilin homologue called DDP1 binds to centromeres, and loss of DDP1 leads to chromosome segregation defects due to impaired pericentromeric heterochromatin formation (41, 42). DDP1 is important for methylation of H3K9 by recruiting Suv39h1 (suppressor of variegation 3-9 homolog 1) to chromatin and helps in recruitment of HP1 (43). DDP1-deficient *Drosophila* cells show reduced H3K9 methylation and mislocalization of HP1 (44). Furthermore, overexpression of DDP1 restores the nuclear segregation and cell morphology defects in Scp160 deletion mutant in budding yeast (41). These studies suggest that regulation of heterochromatin-mediated gene silencing by vigilin is conserved across species and works by maintaining the levels of repressive H3K9 methylation. Heterochromatin organization has been shown to regulate chromatin topology and genome organization (32, 45). Heterochromatin domains are spatially organized within the nucleus, and this domain architecture regulates 3D genome organization and gene expression (46, 47). Since vigilin is important for heterochromatin-mediated gene silencing, it would be interesting to investigate whether vigilin regulates higher-order chromatin organization. Furthermore, proper heterochromatin organization is crucial for stability of repetitive elements like centromeres and telomers; dissecting the importance of vigilin in maintaining the stability of centromeres and telomers would be an interesting area to explore. Vigilin has been found to interact with CTCF, a master regulator of genome organization. Vigilin–CTCF interaction is crucial for CTCF-dependent regulation of Igf2 and H19 expression (24, 48). It would be interesting to dissect how the CTCF–vigilin complex regulates heterochromatin architecture and higher-order genome organization. Vigilin has also been shown to be a key regulator of hepatic apolipoprotein B/Apob mRNA translation (49). Vigilin binds to CU-rich regions of Apob mRNA and regulates its translation (49). Furthermore, vigilin levels corelate with lipid accumulation in patients with nonalcoholic fatty liver disease (NAFLD) and nonalcoholic steatohepatitis, and knockdown of vigilin in liver reduces atherosclerotic plaque formation in mice suggesting an important role of vigilin in liver metabolism (49). Understanding how vigilin regulates NAFLD would be an exciting research question to understand the biology of the highly growing metabolic syndrome NAFLD.

## Vigilin in genome stability

**Figure 3:**
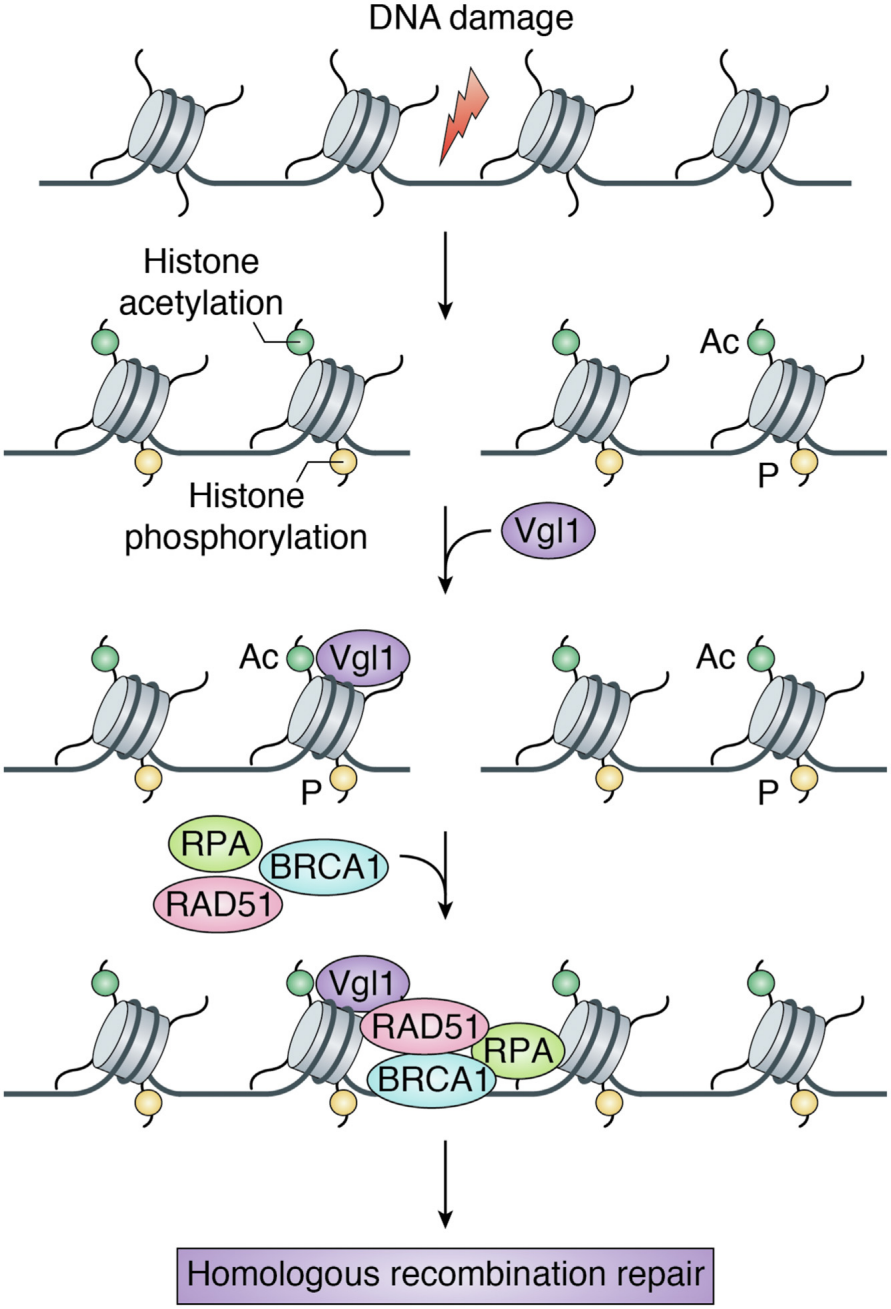
**Vigilin in Genome Stability.** Upon DNA damage, vigilin gets recruited to chromatin in an acetylation-dependent manner. At the break site vigilin interacts with RAD51 and BRCA1 and facilitates their recruitment to DNA DSB sites and replaces 53BP1 to facilitate MRN-mediated DNA end resection. This is followed by RPA loading onto the resulting ssDNA and polymerization by RAD51 to initiate the homologous pairing and strand exchange steps.

Throughout the lifetime of the cell, the genome is constantly exposed to various endogenous and exogenous DNA-damaging agents like reactive oxygen species, base mismatches, UV light, ionizing radiation (IR), and environmental factors (50, 51). DNA repair is critical for normal cellular physiology, and failure to repair results in various diseases like cancer and neurodegenerative disorders (30, 50). Cells have developed intricate mechanisms to recognize the damaged DNA sites and activate processes required for repair of damaged DNA (51, 52). Within the cell nucleus the repair factors have to operate in chromatin context, and chromatin modifications have been shown to play important roles in DNA repair processes (30, 50). Apart from histone modifications, several nonhistone proteins having roles in heterochromatin organization have been shown to be associated with genome stability (53). HP1 gets recruited to DNA damage sites in response to UV-induced DNA damage and is important for efficient repair of damaged DNA (54, 55, 56). Similar to HP1, vigilin has been shown to be important for genome stability across species. Our laboratory has recently found that *S. pombe* cells depleted of vigilin display hypersensitivity to the DNA-damaging agents and show reduced cell survival (27). Like in yeast, loss of vigilin in mammals leads to genomic instability (27). Human cells depleted of vigilin show decreased cell survival in response to DNA-damaging agents like IR and cisplatin and increased spontaneous and IR-induced chromosomal aberrations (27). These defects occurred concomitantly with persistent levels of γ-H2AX (gamma-H2AX), 53BP1 (p53-binding protein 1), and reduced BRCA1 (BReast CAncer gene 1) and RAD51 (DNA repair protein RAD51 homolog 1) foci, indicating defective DNA repair (27). Vigilin interacts with DNA repair proteins like Rad51 and BRCA1, and loss of vigilin impairs their recruitment to chromatin (27). Vigilin-depleted cells show reduced BRCA1 and RAD51 foci and decreased ssDNA formation (27). Vigilin regulates DNA repair through homologous recombination (HR), and loss of vigilin alters pathway choice against error-free homology-directed repair and in favor of error-prone nonhomologous end joining at sites of DNA double-stranded breaks (DSBs) (27). Mechanistically, vigilin gets recruited to DNA damage sites in a histone acetylation–dependent manner and helps in recruitment of repair proteins to DNA damaged sites (27). Treatment of cells with histone acetyltransferase inhibitor, curcumin, reduces vigilin recruitment to chromatin, whereas treatment with histone deacetylase inhibitor trichostatin A increases the occupancy of vigilin to DNA damage sites (27). Furthermore, ectopic expression of vigilin in cells depleted of endogenous vigilin restored cell survival, recruitment of repair proteins to DNA damage sites, and HR defects post irradiation (27). These studies confirm that vigilin is required for DNA DSB repair by the HR pathway. Based on our data, we propose a model for the role of vigilin in DNA repair (Fig. 3); upon DNA damage, vigilin gets recruited to damaged sites in a histone acetylation–dependent manner and helps in recruitment of repair proteins, Rad51/BRCA1 to DNA DSB sites and helps in homology-directed DNA repair. The function of vigilin in DNA repair and its dependence on histone acetylation is contradictory to its role in heterochromatin gene silencing where it promotes repressive H3K9 methylation. The reason for this contradiction could be the proteins that associate with vigilin in context to heterochromatin and DNA repair. Vigilin contains multiple KH domains that are involved in protein–protein interactions. It would be interesting to find the proteins that associate with vigilin at heterochromatin loci and at the DNA break sites. These proteins would shed light on how vigilin regulates heterochromatin formation and DNA damage repair. Vigilin has also been found to be in complex with editing enzyme ADAR1 (adenosine deaminases), DNA-dependent protein kinase (DNA-PK), and RNA helicase A (57). In RNA-dependent manner, association of DNA-PK with this complex leads to phosphorylation of histone H2A-X, an important epigenetic marker in DNA damage response (57, 58, 59). RNA helicase A acts as a binding partner of BRCA1, a tumor suppressor gene involved in many DNA damage repair pathways (60). BRCA1 gets recruited to DNA damage sites through γ-H2AX following DNA damage, and depletion of BRCA1 leads to defects in nonhomologous end joining, transcription-coupled repair, and HR (59, 61). These results suggest that vigilin regulates several pathways in DNA repair process and is essential for repair of damaged DNA. It would be interesting to explore how protein complexes that associate with vigilin regulate its role in various DNA repair pathways.

## Vigilin in cancer

**Figure 4:**
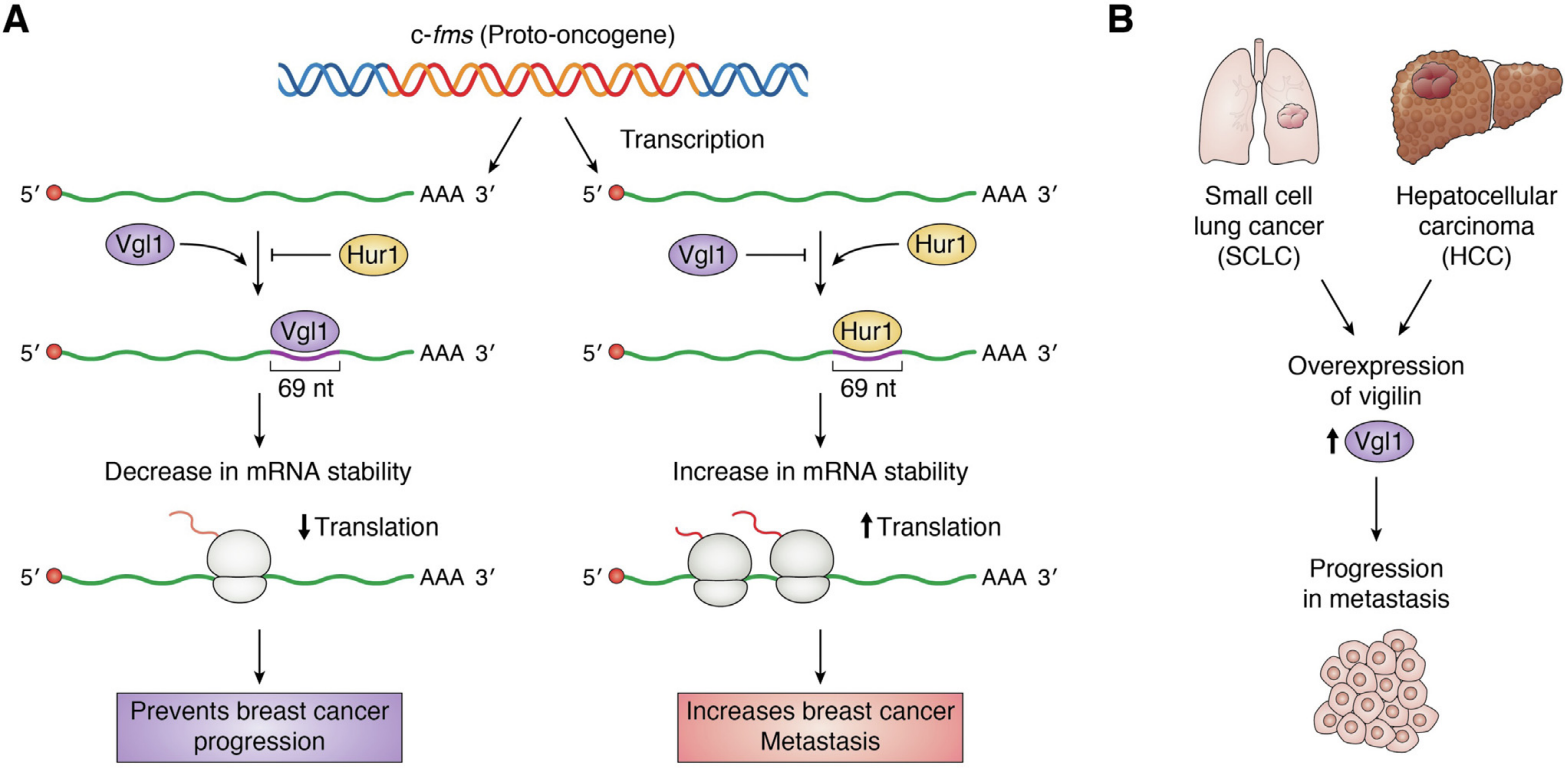
**Vigilin incancer.** (*A*) vigilin competes with HuR (RNA-binding protein) for binding to a novel 69-nt non-ARE-containing 3′ UTR sequence in *c-fms* mRNA. The binding of HuR at 3′UTR of *c-fms* mRNA enhances its stability and translation. Depletion of vigilin increases the degree of breast cancer invasion, while overexpression of vigilin shows decrease in the invasion of breast cancer. *B*, role of vigilin in small cell lung cancer (SCLC) and HCC progression. Overexpressed vigilin in SCLC/HCC tissue samples promotes growth and metastasis of SCLC cells by promoting G1/S cell cycle transition.

Vigilin has been associated with various cancers and found to be upregulated in gastric cancer, prostate cancer, leukemia, and ovarian cancer cells (62). Vigilin controls expression of *c-fms*, a proto-oncogene (which encodes the tyrosine kinase receptor for CSF-1), by decreasing its mRNA half-life and inhibits its translation (63). Vigilin regulates *c-fms* by competing with HuR (Human antigen R) for binding to a novel 69-nt non-ARE-containing 3′ UTR sequence in *c-fms* mRNA (63, 64). HuR, encoded by ELAVL1 (embryonic lethal, abnormal vision, *Drosophila*-like 1) binds at the 3′UTR of *c-fms* mRNA and enhances mRNA stability and translation (63, 64). Knockdown of vigilin or overexpression of HuR increased the degree of breast cancer invasion, while overexpression of vigilin showed a decrease in the invasion of breast cancer (63). *c-fms* proto-oncogene and colony stimulating factor-1 play an important role in the development and progression of breast cancers. *c-fms* expression is high in metastatic breast cancer cells and is strongly associated with lymph node metastasis and poor survival of patients with breast cancer (65, 66, 67). It has been shown that ectopic expression of vigilin in breast cancer cells leads to downregulation of *c-fms* (63). Furthermore, metastatic breast cancer cells show decreased levels of vigilin compared with nontumorigenic epithelial breast cells suggesting the role of vigilin in invasive characters of breast cancer cells (63). Vigilin overexpression has been shown to be associated with hepatocellular carcinoma (HCC), the most common type of primary liver cancer, and occurs most often in people with chronic liver diseases, such as cirrhosis, and is the leading cause of liver cancer–related deaths worldwide (68). Increased vigilin levels have been found in human HCC tissue samples, and degree of expression increases from liver cirrhosis to HCC (69). Vigilin overexpression is important for HCC cell proliferation and tumorigenesis, and deletion of vigilin leads to reduced HCC cell proliferation (69). Furthermore, knockdown of vigilin inhibits the growth of BEL7402 cell–derived xenograft tumors in nude mice by decreasing the proliferation of the BEL7402 HCC cells (69). Vigilin also plays an important role in small cell lung cancer (SCLC) progression (70). Vigilin has been found to be overexpressed in SCLC tissue samples and promotes growth and metastasis of SCLC cells by promoting G1/S cell cycle transition (70). Depletion of vigilin leads to inhibition of SCLC proliferation (70). So, in a context-dependent manner and depending on the type of cancer, vigilin may act as either tumor suppressor or promoter of tumorigenesis (Fig. 4). It will be interesting to uncover the signaling events that direct vigilin toward these two opposing processes. We speculate that posttranslational modifications of vigilin might regulate its switch as tumor suppressor or promoter of tumorigenesis.

## Vigilin in autism spectrum disorders

**Figure 5:**
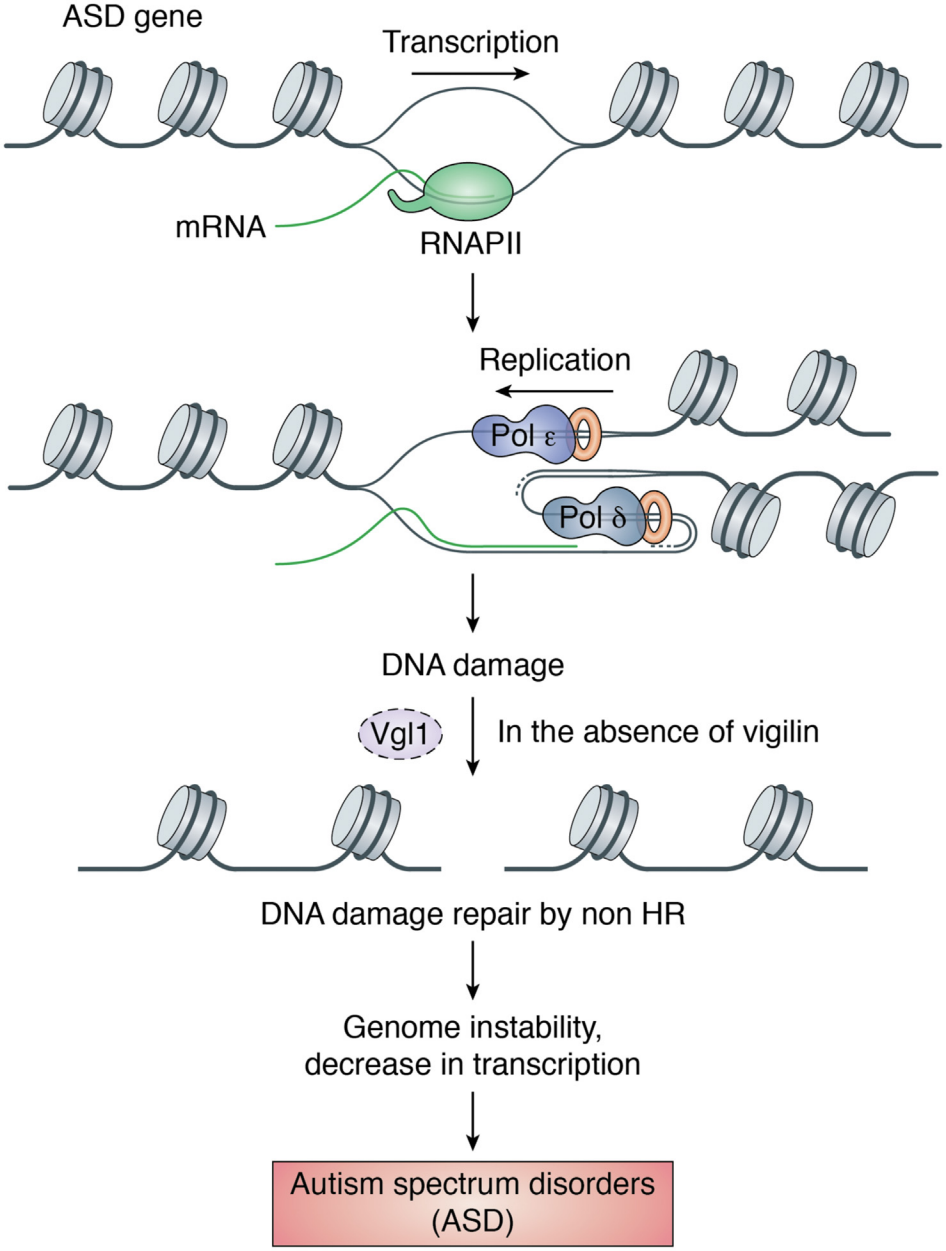
**Vigilin in Autism spectrum disorders.** Model depicting the role for appropriate vigilin levels in preventing genomic instability and reduced transcription at sites of topological conflicts between transcription and replication in ASD.

Autism is a common childhood neurodevelopmental disorder with reduced social cognition and social perception, executive dysfunction, and atypical perceptual and information processing (71). In autism, higher-order association areas of the brain that normally connect to the frontal lobe are partially disconnected during development (72). Vigilin haploinsufficiency has been shown to be associated with autism-related disorders (26). In autism and brachymetaphalangy, a 2q37 deletion syndrome causes downregulation of vigilin, which results in the Albright hereditary osteodystrophy-like phenotype characterized by developmental delay, obesity, short stature, mental retardation, and brachymetaphalangy (26). Expression analysis of vigilin in lymphoblastoid cell lines derived from blood cells of autistic individuals shows considerable downregulation in patient cell lines compared with healthy controls. Vigilin also shows considerable structural similarity with fragile X mental retardation 1 protein (FMR1) as both contain KH domains, which are involved in RNA binding (73). Impaired FMR1 function due to addition of trinucleotide repeat expansion in the promoter region causes fragile-X syndrome, a mental retardation condition (74, 75). Like FMR1, vigilin is involved in transport of mRNA to cytoplasm, and altered mRNA transport due to haploinsufficiency of vigilin could be the possible link between vigilin and autism spectrum disorders (ASDs) (73). Furthermore, fibroblasts from ASD-affected infants and toddlers reprogrammed into neural progenitor cells (NPCs) show increased cell proliferation (76). These NPCs show increased levels of DNA DSBs particularly in long genes (>0.5 Mb) having roles in cell migration and apical polarity (77). Owing to increased DNA damage, these long genes show reduced expression with limited synapsis. Furthermore, these NPCs show increased γ-H2AX levels and defective origin firing (origin firing is DNA replication that initiates at specialized start sites) (77). Depletion of vigilin in other cell lines show similar phenotype as observed in ASD-derived NPCs suggesting that the ASD-related phenotype could be due to vigilin haploinsufficiency (26). Long genes highly expressed in the brain are characterized by extremely long and asymmetric low complexity sequences formed by uninterrupted purine (or pyrimidine) runs on one DNA strand and conversely, pyrimidines on the complementary strand (R•Y tracts), often exceeding 250 bases (78). These extended R•Y sequences are known to form three-stranded nucleic acid structures, whereby homopurine bases with mirror repeat symmetry engage simultaneously in Watson–Crick base pairing with a matching homopyrimidine strand and in Hoogsteen-type pairing with a homopurine or homopyrimidine tract, giving rise to pure DNA or hybrid RNA–DNA complexes (79, 80). As with other DNA secondary structures, triplexes may block replication and transcription, trigger DSBs, and induce a DNA damage response, leading to genetic instability (81, 82, 83, 84, 85). In ASD-derived NPCs, DSB density correlates positively with gene expression levels, supporting the view that replication stress arises from head-on collision of the replication machinery with a stalled transcriptional apparatus, which usually occurs at persistent DNA–RNA hybrids (R-loops) and other structural impediments in the DNA template (86, 87, 88). Of the 37 DSB hotspots identified in the ASD-derived NPCs, 36 occurred within genes, 30 of which were longer than 800 kb, a threshold above which transcription–replication conflicts are inevitable, thereby increasing the liability to genomic alterations such as copy number variants and chromosomal fragile sites (89, 90). By intersecting the genomic coordinates of the 36 DSB hotspots in ASD-derived NPCs with those of R•Y longer than 250 bp, we noted that the R•Y tracts fell within the hotspot’s intervals in five genes and they fell within 0.1 Mb in two other genes. Considering that there are 228 genes harboring R•Y tracts longer than 250 bp genome-wide, the number of genes in ASD-derived NPCs with DSB hotspots containing long R•Y is greater than expected by chance (*p* < 0.00001). Therefore, it is expected that long R•Y sequences will commonly create topological conflicts during the simultaneous transcription and replication in the longest genes in the brain, likely by forming DNA–RNA triplexes behind an RNA Pol II complex (Fig. 5), which are favored by the local accumulation of negative supercoiling and intrinsically high stability (84). Once formed, these structures oppose the progression of a subsequent RNA Pol II complex (91) and will block a DNA Pol complex approaching from the opposite direction (Fig. 5). Although a stalled RNA Pol II complex will prevent the use of a converging replication fork to restart replication, recombination-dependent repair provides a means to complete faithful replication in these long genes (86, 92, 93). However, if HR factors are lacking or in limiting supply, as with *HDLBP* (high-density lipoprotein–binding protein) haploinsufficiency, low replicating regions and regions with sparse origin densities, which include these extremely long genes (90), can accumulate unresolved replication intermediates that will block transcription and persist into mitosis, where they will be resolved by structure-specific nucleases and repaired by error-prone break-induced replication (92, 93) (Fig. 5). Thus, cells depleted of vigilin have a higher frequency of spontaneous chromosome damage, implicating the role of vigilin in ASDs. We postulate a critical dependence for optimal HR activity in the resolution of transcription–replication conflicts caused by complex nucleic acid structures, exemplified here by DNA–RNA triplexes formed by some of the longest R•Y tracts found in the human genome. Testing this hypothesis and other known functions of vigilin will be key to gathering sufficient knowledge on ASD pathology to implement guided therapeutic strategies.

## Conclusions and future perspectives

Vigilin, an evolutionarily conserved KH domain–containing RBP, plays important role in many biological processes like chromosome segregation, heterochromatin organization, mRNA stability, RNA transport and metabolism, and DNA damage repair. It has different functions in the cytoplasm and nucleus. Cytoplasmic vigilin is associated with mRNA stability and translation, whereas nuclear vigilin plays a role in heterochromatin formation, chromosome segregation, and DNA repair. How the nuclear and cytoplasmic functions of vigilin are regulated is not known.

It would be interesting to find the domain structure of vigilin and how it contributes to its localization and function. We previously reported that vigilin is essential for heterochromatin gene silencing at centromeres and telomeres; it would be interesting to investigate whether vigilin regulates 3D genome organization. Proper heterochromatin organization is crucial for stability of repetitive DNA elements like centromeres and telomers; dissecting the importance of vigilin in maintaining the stability of centromeres and telomers would be an interesting area to explore. Vigilin has been shown to be essential for DNA damage repair and regulates both HR and nonhomologous end joining pathways. It would be interesting to explore how protein complexes that associate with vigilin regulate its role in various DNA repair pathways. Vigilin haploinsufficiency has been shown to be associated with autism-related disorders. Vigilin also shows considerable structural similarity with FMR1 as both contain KH domains. Vigilin regulates transport of mRNA and altered mRNA transport due to downregulation of vigilin could be the possible link between vigilin and ADS. Fibroblasts from ASD-affected infants and toddlers reprogrammed into NPCs showed increased levels of DNA DSBs particularly in long genes having roles in cell migration and apical polarity. Due to accumulated DNA double strand breaks, these long genes show reduced expression with limited synapsis possibly due to vigilin haploinsufficiency. It will be very important to elucidate the mechanisms by which vigilin contributes to various autism-associated disorders. Vigilin levels have been shown to corelate with lipid accumulation in patients with NAFLD and nonalcoholic steatohepatitis. Depletion of vigilin in liver reduces atherosclerotic plaque formation in mice suggesting an important role of vigilin in liver metabolism. Understanding how vigilin regulates NAFLD would be an exciting research question to understand the biology of the highly growing metabolic syndrome NAFLD. Despite various reports on vigilin function, we still do not have a clear understanding of how these processes are mechanistically regulated and how a single RBP regulates several biological functions. Vigilin can act as a recruiting factor, a scaffolding entity, or a sequence-specific determinant in regulating these processes. It would be interesting to explore whether other KH domain–containing RBPs function like vigilin and have roles in gene silencing, DNA repair, and their association with various diseases.

## Conflict of interest

The authors declare that they have no conflicts of interest with the contents of this article.
